# The Utility of ADC First-Order Histogram Features for the Prediction of Metachronous Metastases in Rectal Cancer: A Preliminary Study

**DOI:** 10.3390/biology11030452

**Published:** 2022-03-16

**Authors:** Bianca Boca (Petresc), Cosmin Caraiani, Loredana Popa, Andrei Lebovici, Diana Sorina Feier, Carmen Bodale, Mircea Marian Buruian

**Affiliations:** 1 Department of Radiology, “George Emil Palade” University of Medicine, Pharmacy, Science and Technology of Târgu Mureș, 540139 Târgu Mureș, Romania; petresc.bianca@elearn.umfcluj.ro (B.B.); mircea.buruian@umfst.ro (M.M.B.); 2 Department of Radiology, Emergency Clinical County Hospital Cluj-Napoca, 400006 Cluj-Napoca, Romania; andrei.lebovici@umfcluj.ro (A.L.); diana.feier@umfcluj.ro (D.S.F.); 3 Department of Medical Imaging, “Iuliu Hațieganu” University of Medicine and Pharmacy Cluj-Napoca, 400012 Cluj-Napoca, Romania; 4 Department of Radiology, Regional Institute of Gastroenterology and Hepatology “Prof. Dr. Octavian Fodor”, 400158 Cluj-Napoca, Romania; 5 Department of Radiology, “Iuliu Hațieganu” University of Medicine and Pharmacy Cluj-Napoca, 400012 Cluj-Napoca, Romania; 6 Department of Oncology, Amethyst Radiotherapy Center Cluj, 407280 Florești, Romania; carmen.bodale@amethyst-radiotherapy.com; 7 Department of Medical Oncology and Radiotherapy, “Iuliu Hațieganu” University of Medicine and Pharmacy Cluj-Napoca, 400012 Cluj-Napoca, Romania

**Keywords:** rectal cancer, magnetic resonance imaging, apparent diffusion coefficient, metachronous metastases, first-order features, histogram

## Abstract

**Simple Summary:**

Metachronous metastases are the main factors affecting survival in rectal cancer, and 15–25% of patients will develop them at a 5-year follow-up. Early identification of patients with higher risk of developing distant metachronous metastases would help to improve therapeutic protocols and could allow for a more accurate, personalized management. Apparent diffusion coefficient (ADC) represents an MRI quantitative biomarker, which can assess the diffusion characteristics of tissues, depending on the microscopic mobility of water, showing information related to tissue cellularity. First-order histogram-based features statistics describe the frequency distribution of intensity values within a region of interest, revealing microstructural alterations. In our study, we demonstrated that whole-tumor ADC first-order features may provide useful information for the assessment of rectal cancer prognosis, regarding the occurrence of metachronous metastases.

**Abstract:**

This study aims the ability of first-order histogram-based features, derived from ADC maps, to predict the occurrence of metachronous metastases (MM) in rectal cancer. A total of 52 patients with pathologically confirmed rectal adenocarcinoma were retrospectively enrolled and divided into two groups: patients who developed metachronous metastases (n = 15) and patients without metachronous metastases (n = 37). We extracted 17 first-order (FO) histogram-based features from the pretreatment ADC maps. Student’s t-test and Mann–Whitney U test were used for the association between each FO feature and presence of MM. Statistically significant features were combined into a model, using the binary regression logistic method. The receiver operating curve analysis was used to determine the diagnostic performance of the individual parameters and combined model. There were significant differences in ADC 90th percentile, interquartile range, entropy, uniformity, variance, mean absolute deviation, and robust mean absolute deviation in patients with MM, as compared to those without MM (*p* values between 0.002–0.01). The best diagnostic was achieved by the 90th percentile and uniformity, yielding an AUC of 0.74 [95% CI: 0.60–0.8]). The combined model reached an AUC of 0.8 [95% CI: 0.66–0.90]. Our observations point out that ADC first-order features may be useful for predicting metachronous metastases in rectal cancer.

## 1. Introduction

Colorectal cancer is one of the most frequent cancers worldwide, occupying the third position, in terms of incidence and the second place, in terms of mortality [1,2]. Approximately 30–35% of all colorectal tumors are represented by rectal cancers (RC) [2,3]. The current standard treatment for locally advanced rectal cancer (LARC) is neoadjuvant chemoradiotherapy (nCRT), followed by surgery with total mesorectal excision (TME) [4,5]. However, studies report that nCRT has no effect on overall survival [6,7]. Recurrence, in the form of distant metastases, is the main factor affecting the overall survival rate, with 15–25% of patients diagnosed with rectal cancer having developed distant metastases (DM) by the 5-year follow-up [8,9,10]. Therefore, identification of patients with a high risk of developing distant metachronous metastases is a major concern, especially since there is still controversy around the use of adjuvant chemotherapy to improve disease-free survival [11,12,13,14,15,16].

Recent papers have focused on rectal cancer prognosis, investigating both clinicopathological and medical imaging factors as possible predictors of different outcomes. TNM staging, histological grade, extramural vascular invasion (EMVI), neural invasion, circumferential margin (CRM) involvement, pretreatment serum level of carcinoembryonic antigen (CEA), and pathological responses to nCRT have been identified as powerful prognostic factors [17,18,19,20]. Increased intratumoral heterogeneity may also represent a potential prognostic factor [21,22], which can be quantified using different imaging parameters, including several derived from magnetic resonance imaging (MRI) [23].

Diffusion weighted imaging (DWI) is a functional MRI technique, based on the Brownian movement of water molecules in tissues, which can be quantified using apparent diffusion coefficient (ADC). ADC values, expressed in units of mm^2^/s, are displayed as a parametric map (ADC map) that reflects the degree of diffusion of water molecules through different tissues. Studies demonstrated a negative correlation between ADC and tissue characteristics, such as cellularity or proliferation activity [24,25], turning it into a promising biomarker of tumor aggressiveness. As for rectal cancer, many papers have investigated the role of ADC mean values to predict histopathological features and response to nCRT [26]. However, according to a recent meta-analysis, their conclusions are inconsistent [26]. 

First-order statistical parameters are quantitative features derived from the gray-level intensity histograms, which describe the distribution of signal intensity values within a region or volume of interest (VOI). Voxel-based histogram analysis of a VOI can assess the whole tumor volume and offer an objective overview of tumor heterogeneity [23,27]. Recent studies have used histogram analysis in different areas of cancer research, including rectal cancer [28,29,30,31,32]. 

The aim of the present study was to investigate the value of first-order histogram-based features derived from pre-treatment ADC maps for the prediction of distant metachronous metastases of rectal tumors. 

## 2. Materials and Methods

### 2.1. Study Population

The local institutional ethics committee approved this study and informed consent was waived, due to the retrospective nature of the study. A retrospective analysis was conducted in our electronic medical database for patients diagnosed with rectal cancer who underwent an MR examination for initial tumor staging between January 2017 and May 2019. The inclusion criteria were: patients with pathologically confirmed rectal adenocarcinoma, no evidence of metastatic disease at the baseline computed tomography (CT) scan, and oncologic follow-up with CT scan of chest, abdomen, and pelvis, within at least 1 year after the initial tumor staging. The exclusion criteria were as follows: MR examinations with severe artifacts and insufficient quality for proper analysis (15 patients), pathologically confirmed mucinous adenocarcinoma (10 patients), pathologically confirmed anal cancer (3 patients), lack of baseline CT (7 patients), synchronous metastases (16 patients), and incomplete oncologic follow-up data (49 patients). Our final study population consisted of 52 eligible patients. All subjects underwent curative surgery, most of them receiving neoadjuvant chemoradiotherapy before the intervention (48 patients). The clinical follow-up examinations were performed every three months in the first two years and every six months in the following two years and included physical examination, carcinoembryonic antigen testing, and imaging with CT or MRI/PET, when recommended. The study population was divided into two groups: patients who developed metachronous metastases (15 patients) and patients without metachronous metastases (37 patients). Metachronous metastases (MM) were considered any distant metastases, which were diagnosed at a follow-up CT examination, performed after the baseline CT for initial staging. 

### 2.2. Image Acquisition

**Figure 1:**
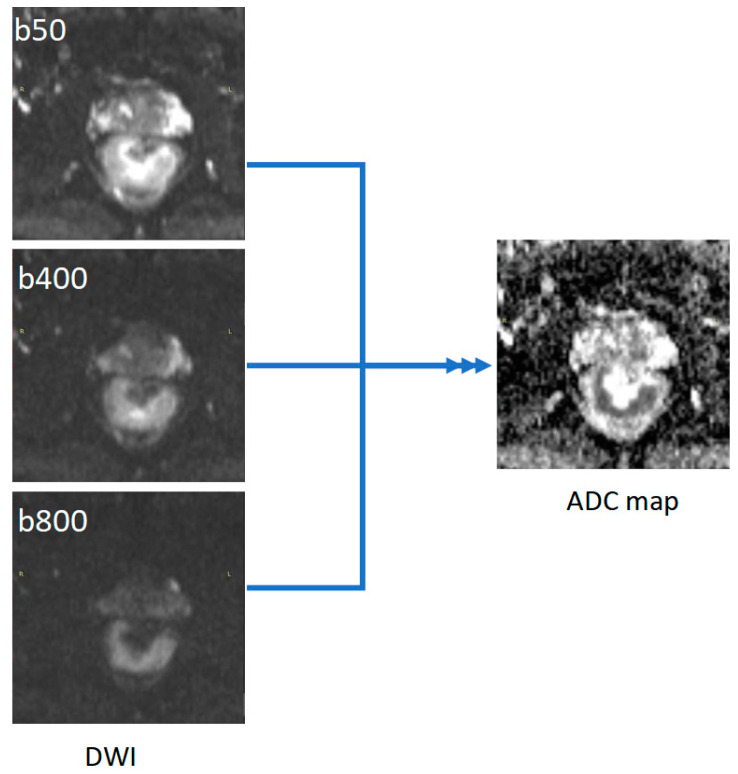
Example of ADC map generated from DWI images with three b values (50, 400, and 800).

All MRI rectal examinations were performed in a single institution, using a 1.5 Tesla MRI scanner (Magnetom Essenza, Siemens AG, Erlangen, Germany), with an eight-channel phased array body coil. The protocol included three T2 weighted turbo spin-echo (TSE) sequences in the sagittal, oblique-axial high-resolution, and oblique coronal high-resolution planes. DWI images were obtained in axial planes using EPI sequences at three b-values (b50, b400, and b800 s/mm^2^). Apparent diffusion coefficient (ADC) maps were automatically generated by the scanner software, using all three b values (Figure 1). ADC values are expressed in 10^−6^ mm^2^/s.

The parameters of the MRI sequences are provided in Table 1. No bowel preparation was received prior to the MRI examination.

### 2.3. Tumor Segmentation and Feature Extraction

Two radiologists (one radiology resident and one senior radiologist with over 10 years of experience in gastrointestinal MRI), blinded to the clinical information and patients’ outcome, independently reviewed the MR images, and delineated the rectal tumors. They manually drew regions of interest (ROIs) along the border of the tumor on each consecutive slice of the ADC maps, covering the whole lesion, resulting in a VOI. Cystic, necrotic, or hemorrhagic areas and artifacts were carefully excluded by referring to T2-weighted and diffusion-weighted images as a guide. The segmentation of the tumors was performed using a designated, open source software 3D Slicer, version 4.10.2 (available at: https://www.slicer.org/, last accessed on 15 December 2021). 

Thereafter, ADC first-order features were automatically extracted from each VOI using the pyRadiomics package, implemented as a plugin into the 3D Slicer software. The following ADC first-order features were extracted from the whole-tumor VOIs: minimum, maximum, mean, median, 10th percentile, 90th percentile, skewness, kurtosis, interquartile range, entropy, energy, uniformity, variance, mean absolute deviation, robust mean absolute deviation, root mean square, and range. A bin width of 25 (standard value in 3D Slicer) was applied before feature extraction. No other preprocessing of the ADC maps was performed. Table 2 provides a brief description for each extracted first-order feature. 

**Figure 2:**
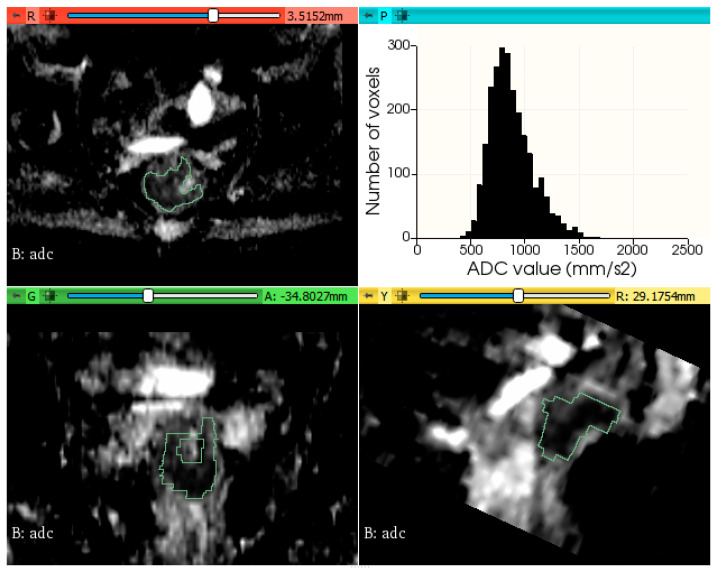
Example of rectal tumor VOI segmentation on ADC map and histogram plot of the ADC values from a patient without metachronous metastases.

**Figure 3:**
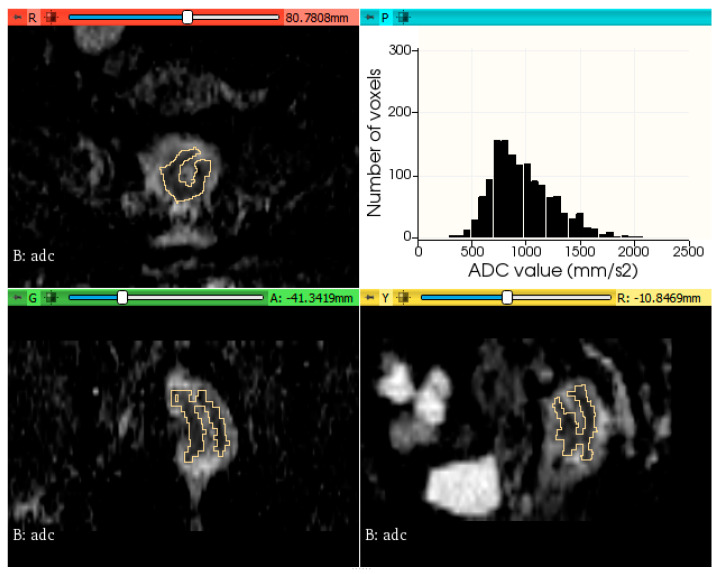
Example of rectal tumor VOI segmentation on ADC map and histogram plot of the ADC values from a patient with metachronous metastases.

The histogram plots of ADC values were generated using a python code, provided in Supplementary File S1. Figure 2 and Figure 3 show two examples of tumor segmentation and histogram plots, representative for each subgroup of patients.

### 2.4. Statistical Analysis

Statistical analysis was performed using commercially available software: MedCalc for Windows, V.14.8 (MedCalc Software, Ostend, Belgium), and SPSS Statistics for Windows, version 18.0 (SPSS Inc., Chicago, IL, USA). Categorical variables are expressed as counts and compared using chi test. Continuous variables are represented as means ± standard deviation. Normality was tested using the Kolmogorov–Smirnov test. 

The inter-reader agreement was evaluated using the intraclass coefficient (ICC) between the features extracted from the radiology resident’s segmentation and senior radiologist’s segmentation. Only features with an ICC ≥ 0.75 were selected for further analysis, and they were averaged between the two observers. 

The differences in ADC histogram metrics between the two groups (non-metastatic vs. metastatic) were evaluated using independent Student’s *t*-test or non-parametric Mann–Whitney U test, in case of non-normally distributed data.

Receiver operating characteristic (ROC) curve analysis was conducted and area under the curve (AUC), sensitivity (Se), specificity (Sp), positive predictive value (PPV), and negative predictive value (NPV) were calculated to evaluate the diagnostic performance of the individual ADC first-order features for the prediction of metachronous metastases. The optimal cut-off value was chosen according to the Youden index. Using binary logistic regression (enter method), the authors created a combined model, which was also evaluated using ROC curve analysis. ROC curves were compared using the method developed by DeLong et al. A *p*-value of <0.05 was considered statistically significant. 

## 3. Results

Among all 52 enrolled patients with rectal cancer, 15 developed distant metachronous metastases. The mean time interval of follow-up was 24 months. The most common site of metastases was the liver (14 patients). Five subjects developed simultaneous liver and lung metastases and one patient was diagnosed with peritoneal carcinomatosis.

A summary of the clinical and histopathological characteristics of the study population is shown in Table 3. No statistically significant differences of any clinical or histopathological variable were observed between the two groups of patients.

The ADC histogram first-order features of the two groups are provided in Table 4. The 90th percentile, interquartile range, entropy, variance, mean absolute deviation, and robust mean absolute deviation were significantly higher among patients with metachronous metastases, compared with the ones without metastases (*p* values ranging between 0.002–0.01). Uniformity was lower in subjects with distant tumor spread. However, there were no significant differences between the two subgroups, regarding the other first-order features. 

Table 5 demonstrates the results of the ROC analysis of ADC parameters for the detection of patients who developed metachronous metastases. The 90th percentile and uniformity showed the best diagnostic performance for predicting the occurrence of metachronous metastases, yielding an AUC of 0.74 [95% CI: 0.60–0.85]. Variance achieved the lowest AUC of 0.65 [95% CI: 0.51–0.78]. The highest sensitivity (80.0%) was achieved by 90th percentile for the cut-off value of 1236.2. Variance reached the highest specificity (86.49%) for the cut-off value of 57046. When comparing the diagnostic performance between individual ADC features, we found a statistically significant difference only between uniformity and variance (*p* = 0.03). Using logistic regression (entry method), we incorporated these seven first-order features into a combined model, which achieved a higher AUC of 0.80 [95% CI: 0.66–0.90]. However, this was only significantly different from the AUC of variance (*p* = 0.04). 

## 4. Discussion

Our study evaluated the potential of histogram metrics, derived from pretreatment ADC maps, to predict the occurrence of metachronous metastases in patients diagnosed with rectal cancer. In the past years, there has been an increased interest in the field of medical imaging texture analysis for lesion characterization, therapy guidance, and tumor prognosis. 

Regarding rectal cancer outcome, the majority of papers focused on predicting tumor response to nCRT, using a single MRI sequence (T2-weighted images or ADC maps) or multiparametric approach [33,34,35]. Only a few studies chose the occurrence of metachronous metastases as the endpoint of their research, most of them obtaining predicting models, based on radiomics features extracted from T2 weighted images [36,37,38]. 

For the current research, we chose to not include all the radiomics features extracted from the tumor, but only first-order features derived from histogram. In spite of the possible advantage of higher-order statistics to better reflect intratumoral heterogeneity, previous studies have demonstrated that, with respect to rectal cancer, first-order texture parameters proved to be more stable and sufficiently robust, being less sensitive to interobserver variability and image processing [39,40]. 

The association between whole-rectal tumor ADC histogram parameters and different clinical pathologic prognostic factors has been the main subject of many papers, however, with conflicting factors [31,32,41,42,43,44,45,46,47,48]. Regarding pT stage, a well-recognized powerful prognostic factor, there are several studies that demonstrate significantly lower values of ADC histogram metrics in tumors with pT1-T2 stages [32,41,49]. Conversely, other investigations found no significant differences in ADC histogram percentiles among different pT stages [50]. Additionally, inconsistent results are reported for the role of pretreatment ADC histogram metrics in predicting treatment response to nCRT. The investigations of Liang et al. [51] and Palmisano et al. [52] obtained AUCs between 0.82 and 0.89 for histogram parameters, such as mean, median, and 75th percentile, to distinguish between pCR and nonPCR, while the studies of Choi et al. [48] and Nougaret et al. [53] found no benefit in any of the ADC histogram metrics for pretreatment prediction of response. The recent meta-analysis of Surov et al. concluded that the mean ADC cannot predict histopathological features and response to nCRT in rectal cancer [26]. Additionally, the results of van Heeswijk et al. [47] and Chidambaram et al. [46] revealed that ADC histogram analyses of rectal cancer were not beneficial to obtaining prognostic information. In our study, we did not find any significant difference in mean ADC between metastatic vs. nonmetastatic tumors. Therefore, our observations support the conclusions of these latter studies, suggesting that ADC mean value has no additional prognostic value. 

However, in this current research, ADC 90th percentile showed the best diagnostic performance in predicting metachronous metastases. Contrary to our expectations, patients who developed MM had significantly higher values of ADC in the 90th percentile. One possible explanation for this finding might be that more aggressive cancers may contain many invisible areas of cystic and necrotic components, which cannot be completely excluded using manual segmentation. Similar results, with higher ADC percentiles, in more aggressive lesions, have also been reported in other oncological investigations [54,55]. 

All the studies mentioned above included in their analysis only ADC mean, histogram percentiles ± skewness, and kurtosis. In addition, we also assessed the role of other first order histogram-based features, such as entropy, uniformity, variance, mean absolute deviation, etc., for the differentiation of tumors, which developed metachronous metastases versus the ones without distant spread. Our results suggest that evaluating all the first-order texture features may bring additional information and improve the prognostic ability of ADC maps. Entropy specifies the randomness in the image values, and it measures the average amount of information required to encode the image values [56]. Uniformity is a measure of the sum of the squares of each intensity value, and it indicates the homogeneity of the image array [56]. A lower value of uniformity, as well as a high value of entropy, indicate greater heterogeneity of the lesion [57]. According to our results, prognostically unfavorable tumors have significantly lower uniformity and higher entropy values and are, therefore, more heterogeneous. Similar findings have been reported in previous studies. Regarding entropy, several authors revealed rectal tumors with higher pT stages showing significantly higher values of ADC entropy, reporting AUCs between 0.67–0.74 [31,42,57,58]. As for uniformity, the results of Meng et al. showed significantly lower ADC uniformity values in rectal tumors, without a complete response to nCRT, obtaining an AUC of 0.69 [59]. Additionally, Lu et al. reported lower uniformity in pT3-T4 rectal cancers, compared to pT1-T2 lesions, with an AUC of 0.63 [57]. In our study, each statistically significant first-order feature achieved an individual AUC between 0.65–0.74 for differentiating MM+ from MM-tumors, while the combined model increased the prognostic efficacy, yielding a moderate AUC of 0.8. 

To the best of our knowledge, this is the first study to investigate the association of all first-order texture features, derived from ADC maps, with the occurrence of metachronous metastases. The research of Yu et al. evaluated the correlation between ADC histogram parameters and distant metastases (DM) from rectal cancer; however, they considered DM as both synchronous and metachronous metastases, and they did not include in their analysis all the first-order histogram features [60]. Conversely to our results, they found significantly higher kurtosis values of tumors with DM. Another paper of Chidambaram et al. reported a significant correlation between skewness and disease progression; however, in their investigation, kurtosis did not differ significantly between the different subgroups [46]. This discrepancy between studies might have resulted from the small number of patients included. 

The current study has some limitations. First of all, its retrospective nature might have led to unintended selection bias. Secondly, many patients did not undergo a proper oncological follow-up, reducing our study population. The MRI examinations were selected from a single center, lacking multicenter validation. Additionally, we included only first-order features, derived from ADC maps, and the role of other imaging sequences may be investigated in future research. 

## 5. Conclusions

Our study suggests that first-order histograms that derive features extracted from pre-treatment ADC maps might represent a potential imaging biomarker to predict metachronous metastases of rectal cancer. However, these results were obtained using a small population and need to be externally validated in larger, multicentric, prospective studies.

## Figures and Tables

**Table 1:** MRI parameters.

MRI Parameter	TSE T2-Weighted Image			DWI
	Sagittal	HR Coronal Oblique	HR Axial Oblique	
TR (ms)	3500	3500	4000	5800
TE (ms)	91	91	80	96
Slice no	28	25	25	30
Bandwidth (Hz/pixel)	391	391	391	1132
FOV (mm)	220	220	200	250
Slice thickness (mm)	3	4	3	4
Matrix	256 × 256	256 × 256	256 × 256	136 × 160
Acquisition time (min)	4	5.5	6	4.5

**Table 2:** Description of ADC first-order histogram-based features.

ADC First-Order Histogram Feature	Description
Minimum	The minimum ADC value within the VOI.
Maximum	The maximum ADC value within the VOI.
Mean	The average ADC value within the VOI.
Median	The ADC value below 50% of all ADC voxel values lie.
10th percentile	The ADC value below 10% of all ADC voxel values lie.
90th percentile	The ADC value below 90% of all ADC voxel values lie.
Skewness	Measures the asymmetry of the distribution of ADC values around the mean value.
Kurtosis	Measures the ‘peakedness’ of the distribution of ADC values within the VOI.
Interquartile range	Measures the spread of the distribution of ADC values, defined as the difference between 75th and 25th percentile.
Entropy	Measures the inherent randomness in the ADC values within the VOI.
Energy	Measures the squared magnitude of ADC values within the VOI.
Uniformity	Measures the homogeneity in the ADC values within the VOI.
Variance	Measures squared distances of each ADC value of a histogram from the mean.
Mean absolute deviation	Mean distance of all ADC values from the mean value of the image array.
Robust mean absolute deviation	Mean distance of all ADC values from the mean value calculated on the subset of image array with ADC in between, or equal to the 10th and 90th percentile.
Range	Measures difference between the highest and lowest ADC values.
RootMeanSquared	Square root of the mean of all the squared ADC values of the histogram. This feature is another measure of the magnitude of a histogram.

**Table 3:** Clinical and histopathological characteristics of the study population.

Variable	Non Metastases (MM-) Group (n = 37)	Metachronous Metastases (MM+) Group (n = 15)	*p* Value
Age (years) *	59.27 ± 11.11	61.87 ± 9.85	0.43
Gender			0.29
Male	27	8	
Female	10	7	
Tumor length (mm) *	58.22 ± 19.74	53.93 ± 18.57	0.47
Tumor differentiation grade			0.41
G1–G2	36	13	
G3	1	2	
Clinical tumor stage (cT)			
T2	9	3	0.98
T3–T4	28	12	
Clinical nodal stage (cN)			0.93
N1	17	6	
N2	20	9	
Mesorectal fascia (MRF) involvement			0.80
Positive	29	12	
Negative	8	3	
Extramural vascular invasion (EMVI)			0.95
Positive	4	1	
Negative	33	14	
Pathological tumor stage (pT)			0.15
pT0-pT2	17	3	
pT3	20	12	
Pathological nodal stage (pN)			0.30
pN0	27	8	
pN1-N2	10	7	

* Results are presented as mean ± standard deviation or number.

**Table 4:** Association of ADC first-order features and the presence of metachronous metastases.

ADC First-Order Feature	MM-	MM+	*p* Value
Minimum ^^^	310.84 ± 193.73	243.87 ± 210.26	0.28
Maximum ^^^	1972.22 ± 284.54	2047.13 ± 294.47	0.40
Mean ^^^	927.20 ± 100.45	974.48 ± 93.91	0.12
Median ^^^	901.96 ± 98.87	949.07 ± 104.04	0.13
10th percentile ^^^	679.14 ± 101.11	694.65 ± 91.58	0.61
90th percentile ^^^	1210.20 ± 112.90	1293.60 ± 103.65	0.02 *
Skewness	0.72 ± 0.41	0.60 ± 0.29	0.32
Kurtosis	4.52 ± 1.11	3.90 ± 0.74	0.05
Interquartile Range	269.62 ± 33.63	308.42 ± 51.64	0.002 *
Entropy	5.04 ± 0.15	5.20 ± 0.22	0.005 *
Energy	1,465,303,600.05 ± 1,764,343,232.40	1,814,657,493.13 ± 1,913,810,213.47	0.53
Uniformity	0.037 ± 0.004	0.032 ± 0.005	0.004 *
Variance	48,432.81 ± 11,167.16	59,287.71 ± 18,590.48	0.01 *
Mean absolute deviation	168.38 ± 19.22	188.70 ± 29.88	0.005 *
Robust mean Absolute deviation	112.90 ± 13.60	129.40 ± 21.38	0.002 *
Range	1661.38 ± 340.22	1803.27 ± 467.76	0.23
RootMeanSquared	953.14 ± 98.66	1004.59 ± 92.28	0.08

* Statistically significant *p* < 0.05. ^^^ The unit of values is 10^−6^ mm^2^/s.

**Table 5:** Diagnostic performance of ADC first-order features for predicting metachronous metastases.

ADC First-Order Feature	Cut-Off Value	AUC [95% CI]	Se [95% CI]	Sp [95% CI]	PPV [95% CI]	NPV [95% CI]
90th percentile	>1236.2 *	0.74 [0.60–0.85]	80.0 [51.9–95.7]	64.86 [47.5–79.8]	48.0 [27.8–68.7]	88.9 [70.8–97.6]
Interquartile range	>287.25	0.72 [0.58–0.83]	73.33 [38.4–88.2]	75.68 [58.8–88.2]	52.6 [28.9–75.6]	84.8 [68.1–94.9]
Entropy	>5.125	0.7 [0.56–0.82]	60.00 [32.3–83.7]	83.78 [68.0–93.8]	60.00 [32.3–83.7]	83.8 [68.0–93.8]
Uniformity	≤0.0344	0.74 [0.60–0.85]	73.33 [44.9–92.2]	78.38 [61.8–90.2]	57.9 [33.5–79.7]	87.9 [71.8–96.6]
Variance	>57046	0.65 [0.51–0.78]	53.33 [26.6–78.7]	86.49 [71.2–95.5]	61.5 [31.6–86.1]	82.1 [66.5–92.5]
Mean absolute deviation	>175.89	0.70 [0.55–0.82]	66.67 [38.4–88.2]	78.38 [61.8–90.2]	55.6 [30.8–78.5]	85.3 [68.9–95.0]
Robust mean absolute deviation	>119.2689	0.73 [0.59–0.84]	73.33 [44.9–92.2]	78.38 [61.8–90.2]	57.9 [33.5–79.7]	87.9 [71.8–96.6]

* The unit of values is 10^−6^ mm^2^/s.

## Data Availability

Data available on request due to privacy restrictions.

## Supplementary Materials

The following supporting information can be downloaded at: https://www.mdpi.com/article/10.3390/biology11030452/s1, Supplementary File S1: Python code for generation of histogram plot.
