# Supplementary material for: The Utility of ADC First-Order Histogram Features for the Prediction of Metachronous Metastases in Rectal Cancer: A Preliminary Study
Source: Biology (Basel). 2022 Mar 16;11(3):452. doi: 10.3390/biology11030452 (PMC8945327; doi:10.3390/biology11030452)
Supplement: Supplementary file 1 [file biology-11-00452-s001.zip › biology-1560278-supplementary.pdf]

Supplementary Material

Python code for generation of histogram plot

```
masterVolumeNode = getNode ('adc')
segmentationNode = getNode('Segmentation')
labelValue = 1
labelmapVolumeNode = slicer.mrmlScene.AddNewNodeByClass('vtkMRMLLabelMapVolumeNode')
slicer.modules.segmentations.logic().ExportVisibleSegmentsToLabelmapNode(segmentationNode,
labelmapVolumeNode, masterVolumeNode)
volumeArray = slicer.util.arrayFromVolume(masterVolumeNode)
labelArray = slicer.util.arrayFromVolume(labelmapVolumeNode)
segmentVoxels = volumeArray[labelArray==labelValue]
import numpy as np
histogram = np.histogram(segmentVoxels, bins=25)
slicer.util.plot(histogram,xColumnIndex = 1)
```

'adc' = the name of the volume node used for feature extraction

'Segmentation' = the name of the segment
